# Comparative Genome Analysis of Two Isolates of the Fish Pathogen *Piscirickettsia salmonis* from Different Hosts Reveals Major Differences in Virulence-Associated Secretion Systems

**DOI:** 10.1128/genomeA.01219-14

**Published:** 2014-12-18

**Authors:** Harry Bohle, Patricio Henríquez, Horst Grothusen, Esteban Navas, Alvaro Sandoval, Fernando Bustamante, Patricio Bustos, Marcos Mancilla

**Affiliations:** Laboratorio de Diagnóstico y Biotecnología, ADL Diagnostic Chile Ltda., Puerto Montt, Chile

## Abstract

Outbreaks caused by *Piscirickettsia salmonis* are one of the major threats to the sustainability of the Chilean salmon industry. We report here the annotated draft genomes of two *P. salmonis* isolates recovered from different salmonid species. A comparative analysis showed that the number of virulence-associated secretion systems constitutes a main genomic difference.

## GENOME ANNOUNCEMENT

*Piscirickettsia salmonis* is a Gram-negative bacterium and the causative agent of a systemic infection known as piscirickettsiosis (1). The disease is a main concern due to its high prevalence in marine rearing areas; it is thus responsible for huge economic losses (2). *P. salmonis* was isolated for the first time in coho salmon (*Oncorhynchus kisutch*) in 1989, being described as an obligate intracellular parasite (3), a fact that delayed the study of its pathogenesis and the development of vaccines against the disease. However, the introduction of solid medium (4) allowed for a demonstration of the facultative intracellular nature of the pathogen and sequencing of the *P. salmonis* strains LF-89 (5) and AUSTRAL-005 (6).

An early phylogenetic study based on the sequence of the internal transcribed spacer (ITS) placed the *P. salmonis* EM-90 strain, which was isolated from Atlantic salmon (*Salmo salar*), apart from the LF-89-like isolates collected from three salmonid species (7). Using a similar approach, we identified EM-90-like isolates in Atlantic salmon only (our unpublished data), suggesting a common genogroup. These isolates were found to be susceptible to quinolones presenting a particular *gyrA* genotype (8). Moreover, their mucoid texture is quite different from the “sticky” phenotype shown by LF-89-like colonies. In order to decipher the genetic basis of such phenotypic traits, we sequenced two *P. salmonis* isolates: A1-15972 (EM-90-like) and B1-32597 (LF-89-like), recovered in 2010 and 2012 from Atlantic and coho salmon, respectively. Sequencing was performed at Macrogen, Inc. (Seoul, South Korea) on the Illumina HiSeq 2000 platform. A total of 103,741,344 and 116,881,806 reads (100 bp) were *de novo* assembled using Platanus version 1.2.1 (9), RepARK version 1.2.2 (10), and Novoalign version 3.02 into the draft genomes of 3,138,697 bp (360 contigs; *N*_50_, 26,215) and 3,461,332 bp (308 contigs; *N*_50_, 28,873) for A1-15972 and B1-32597, respectively. The genomes had a similar G+C content of ~38.0%. The assembled data were annotated with Prodigal (11), Blast2GO (12), tRNAscan (13), and RNAmmer (14), which predicted 3,478 coding sequences (CDSs), 12 rRNAs, and 56 tRNAs for A1-15972, and 3,840 CDSs, 12 rRNAs, and 56 tRNAs for B1-32597. A comparison using MUMmer version 3.23 (15) and CLC Genomic Workbench Version 6.5.1. revealed 123,822 single nucleotide polymorphisms (SNPs) and 998 insertions/deletions (indels), as well as 247 specific genes for A1-15972 and 351 for B1-32597.

Type IV secretion systems (T4SSs) are well-known virulence-associated multiprotein complexes (16). Recently, the expression of T4SS-related genes was reported in LF-89 (17). Consistent with this previous work, three T4SSs were found in the B1-32597 genome. Remarkably, a comparative analysis performed with Mauve (18) disclosed that the A1-15972 genome contains two T4SSs, lacking ~30 kb that bear a complete T4SS in B1-32597. A ~20-kb indel encoding *tra* genes was detected in B1-32597 only. Such indels may be related to the narrow host range exhibited by A1-like isolates. Regarding the colony phenotype, gross differences can be linked to polymorphisms on lipopolysaccharide (LPS) genes, specifically those encoding the O antigen (19). Interestingly, four glycosyltransferase genes were deleted in A1-15972. Additionally, the A1-15972 genome harbors more LPS-related kinase genes than those found in B1-32597. Further functional characterization is required to prove these hypotheses.

The new sequences will allow a more comprehensive phylogenetic analysis of *P. salmonis*.

### Nucleotide sequence accession numbers.

The sequences of A1-15972 and B1-32597 are part of a sequencing project, which has been deposited at DDBJ/EMBL/GenBank under the accession numbers JRAV00000000 and JRAD00000000, respectively. The versions described in this paper are the second versions, JRAV02000000 and JRAD02000000, respectively.
